# Arteriovenous fistula following kidney biopsy

**DOI:** 10.4103/0971-4065.42348

**Published:** 2008-04

**Authors:** D. Sreebhushan Raju, S. Rammurti

**Affiliations:** Department of Nephrology, Nizam's Institute of Medical Sciences, Hyderabad, India; 1 Department of Radiology, Nizam's Institute of Medical Sciences, Hyderabad, India

A 35-year-old man was admitted to our hospital with loss of appetite for two months and difficulty in breathing for one week. He was blind since birth and has no family history of renal disease. He did not have any auditory or musculoskeletal abnormality. Clinical examination revealed the following: blood urea, 232 mg/dL; serum creatinine, 10.5 mg/dL; sodium, 144 meq/L; potassium, 5.8 meq/L; hemoglobin, 8.8 gm/dL. An ultrasonography revealed the sizes of the right and left kidneys to be 9.2 × 4.2 and 9.4 × 4.5 cm, respectively. He was administered acute peritoneal dialysis for three days, and percutaneous renal biopsy was performed on the fourth day under ultrasound guidance.

**Fig. 1:**
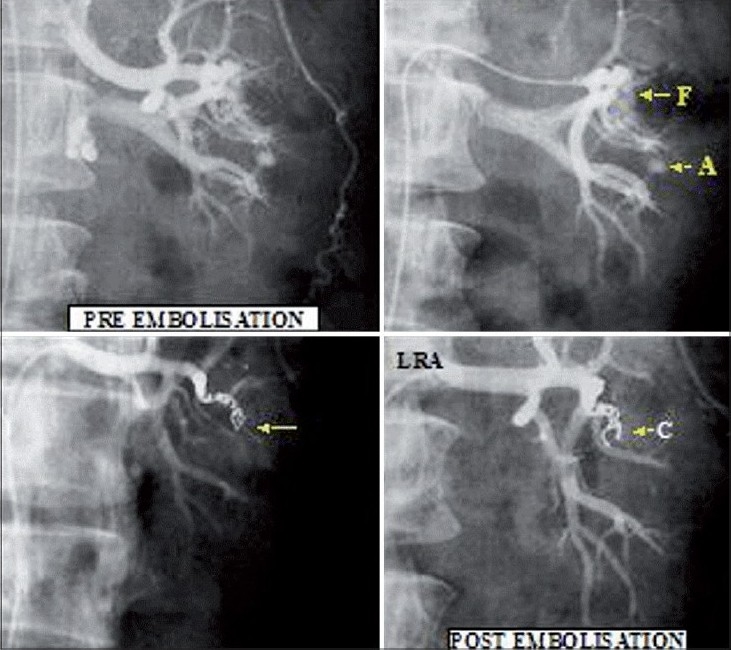
Upper panel: Left, fistulous communication between arterial and venous system; Right, showing aneurysm (F: fistula and A: aneurysm) Lower panel: Left, fistula closed; Right, check angiogram showing an absence of AVF (C: Coils)

On the day following renal biopsy, the patient complained of hematuria, which remained persistent for four days. Although his blood pressure was stable, a drop in hemoglobin was observed. He was initiated on hemodialysis via double lumen jugular catheter and administered packed cell transfusion. Color Doppler of kidneys was suggestive of an arteriovenous fistula (AVF). After a few sessions of hemodialysis, a diagnostic renal angiogram was performed with 4F hydrophilic catheter (Simmons's), which revealed an AVF involving the mid polar segmental vessels [Fig. 1]. Super selective cannulation was performed with 2.7/2.9 F (Progreat, Terumo Corporation, Japan) microcatheter introduced coaxially through a 4F guiding catheter. Platinum coils (0.018” Cook, USA) were deployed to occlude the fistulous communication. A check angiogram following coil embolization revealed an obliteration of the flow across the fistulous communication.

The patient became asymptomatic and was continued on hemodialysis. Biopsy was suggestive of end-stage renal disease with significant tubulointerstitial damage and all the glomeruli were sclerosed. He was discharged and later lost to follow-up.

Renal biopsy is an important intervention that provides information regarding the diagnosis, management and prognosis of patients with renal disease. The procedure of renal biopsy is simple and safe in expert hands.^1^ Use of biopsy gun and real time ultrasound guidance has further enhanced the safety of this procedure. However, renal biopsy does have complications. Asymptomatic hematomas (30-90%) and micro or macroscopic hematuria (2-10%) to life-threatening complications that may require nephrectomy have been reported. Bleeding requiring transfusion or other clinical intervention occurs in only 1-6% of patients. The incidence of AVF appears to be low and has been infrequently reported in large series of biopsies although a number of individual reports also exist. AVFs can be demonstrated by arteriography in approximately 15% (range: 1-18%) of patients, and the frequency is higher in renal allografts (7% *vs* 3% in native kidneys).^2^ In most cases, they remain asymptomatic and resolve spontaneously (80%) over the next few months. Sometimes they can lead to persistent bleeding or recurrence of bleeding even few months after biopsy, uncontrollable hypertension and/or sudden or gradual deterioration in the renal function.^3^ Life-threatening complications such as high-output cardiac failure and peripheral thromboembolization have also been reported.

Color-coded Doppler imaging can accurately diagnose postrenal biopsy AVF.^4^ Angiography is the gold standard that should be performed whenever clinically indicated for the localization of the AVF. Endovascular intervention is the procedure of choice.^5^ Different technical methods of embolization are available. Successful transcatheter arterial embolization is carried out with a variety of materials, including Gel foam pledgets (Upjohn, Kalamazoo, MI, USA), stainless steel or platinum coils (with or without Dacron fibers) and polyvinyl alcohol particles. The success rate is approximately 88%. The complications of the procedure include renal infarction, hemorrhage and coil migration. Superselective embolization of AVF is safe and effective.^6^ It is important to note that angiographically successful embolization is not always necessarily associated with clinical success. Surgical treatment indicated for AVF is partial or total nephrectomy^7^ or arterial ligation. The technique for renal biopsy should aim at not only obtaining kidney tissue that is technically adequate for diagnosis but also reducing biopsy-induced complications.
